# Comparative hearing outcomes of Stapedotomy and hearing aid rehabilitation in otosclerosis

**DOI:** 10.1007/s00405-025-09754-6

**Published:** 2025-10-18

**Authors:** Andreas Björsne, Ylva Dahlin Redfors, Caterina Finizia

**Affiliations:** 1 Department of Otorhinolaryngology, Head and Neck Surgery, Institute of Clinical Sciences, Sahlgrenska Academy, University of Gothenburg, Gothenburg, Sweden; 2 Department of Otorhinolaryngology, Head and Neck Surgery,Sahlgrenska University Hospital, Region Västra Götaland, Gothenburg, Sweden

**Keywords:** Otosclerosis, Stapedotomy, Hearing aid, Conductive hearing loss, Disability

## Abstract

**Purpose:**

This prospective, non-randomized intervention study aimed to compare hearing outcomes and patient satisfaction between stapedotomy and hearing aid rehabilitation in individuals with untreated otosclerosis.

**Methods:**

A total of 134 adults diagnosed with otosclerosis were recruited from four Swedish clinics. Participants self-selected treatment after receiving standardized information: 91 chose stapedotomy and 43 opted for hearing aids. Hearing outcomes were assessed pre- and one year post-intervention using pure tone audiometry, sound field (SF) warble tones, and speech reception thresholds (SRT) in noise using the Hagerman matrix test. Patient satisfaction was also evaluated.

**Results:**

Baseline audiometric thresholds were similar between the two groups. At follow-up, stapedotomy resulted in greater low-frequency SF threshold improvements at 250 and 500 Hz (*p* < 0.001 and *p* = 0.002, respectively), and significantly better SRT in noise (*p* = 0.004). Subjective satisfaction was high in both groups, but was significantly greater among stapedotomy recipients (*p* = 0.019).

**Conclusion:**

Stapedotomy was associated with better speech-in-noise recognition, low-frequency hearing gain, and patient satisfaction compared to hearing aids in otosclerosis patients, although both treatments were effective. Treatment choice should consider individual clinical profiles, patient preferences, and potential risks, as stapedotomy is associated with more serious adverse effects despite superior auditory outcomes.

## Introduction

Otosclerosis is a disease of the otic capsule characterized by abnormal bone remodeling. It primarily affects the stapes, causing fixation of the stapedial footplate, leading to progressive conductive or mixed hearing loss. The disease usually has an onset around 30 to 40 years of age and can be unilateral or bilateral [1, 2]. The associated hearing loss can be treated either by surgery, which aims to restore sound transmission to the inner ear, or by sound amplification with a hearing aid. The surgical procedure of choice today is stapedotomy, which involves removing the stapes superstructures and creating a small hole in the footplate (fenestration) using a drill or a laser [3–5].

Stapedotomy is considered to be a safe procedure that generally results in successful hearing outcomes with few complications, but it is not completely without risk [5–7]. More pronounced tinnitus and loudness discomfort can occur after the surgery in a few cases, as well as vertigo or dizziness, and, further, there is a small risk of additional hearing loss and even deafness [5, 7–9]. An air conduction hearing aid does not carry the same risks and is often recommended to be tried before surgery and for those who choose not to have surgery [3]. Hearing aid use is further advocated for individuals with sensorineural deafness in the contralateral ear. However, some experience problems with otitis externa, which can be caused by the obstruction of the ear canal or an allergic reaction to the material of the earmold; in severe cases, this can make hearing aid use difficult [10]. An important consideration related to hearing aids is the stigma that can be associated with it, making them an undesirable option for some [11]. Moreover, hearing aids have maintenance needs to work properly. The choice between surgery and hearing aid rehabilitation may also depend on availability and economic factors.

To our knowledge, Molinier et al. [12] have performed the only prospective study comparing hearing aid use and stapedotomy in otosclerosis patients. This study included 20 patients who underwent stapedotomy after a two-month hearing aid trial, i.e., the included patients underwent both treatments in sequence. After stapedotomy, patients had better hearing and performed better in speech audiometry testing compared to after the hearing aid trial and also reported higher quality of life.

Further larger-scale studies comparing treatment outcomes in otosclerosis are warranted. Ideally, such studies should include patient groups who have not previously received either hearing aid amplification or surgical treatment. This approach may help to reduce potential selection bias inherent in sequential study designs, where all participants must desire surgery. In addition, postoperative assessment of hearing improvement after stapes surgery should preferably be performed at least one year following the procedure [13].

The aim of this prospective study is to compare hearing outcomes of stapedotomy and hearing aid intervention in patients with no previous treatment for their otosclerosis.

## Methods

### Participants

**Fig. 1:**
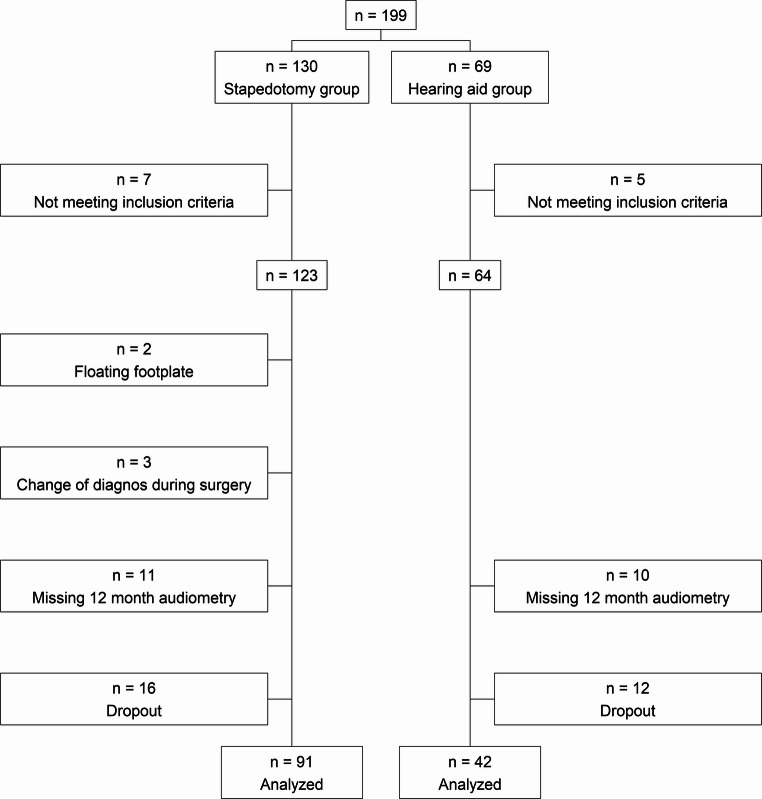
Study flowchart of included participants

This is a prospective, non-randomized intervention study including participants recruited from two university clinics and two county clinics. A total of 133 participants were included in the study, 91 were included in the stapedotomy group, and 42 in the hearing aid group, Fig. 1. All participants were diagnosed with otosclerosis and had no prior treatment for the condition. The diagnosis of otosclerosis was established based on the presence of conductive or mixed hearing loss, accompanied by an acoustic reflex response pattern indicating a fixation of the stapes in the affected ear. The participants met the following inclusion criteria: air conduction (AC) pure tone average for frequencies 500, 1000, 2000 and 4000 Hz (PTA^4^) $\:\ge\:$ 30 dBHL, bone conduction (BC) PTA^4^ $\:\le\:$ 40 dBHL, an air bone gap (ABG) $\:\ge\:$ 20 dB at 500 and 1000 Hz, and not having any severe chronic health issues. Only adults (20–65 years) were included. A good knowledge of the Swedish language was also required.

Participants were thoroughly informed about the treatment options, i.e., restorative surgery or acoustic amplification with a hearing aid, and the decision of treatment was then made by each participant. In Sweden, both restorative surgery and rehabilitation with hearing aids are publicly funded, with the financial burden for the patient being relatively similar for the two treatment options. Only unilateral treatments were considered for this study, and the ear with poorer hearing was treated. In the participating clinics, stapedotomies are performed sequentially, generally one year or more apart in cases of bilateral otosclerosis. However, hearing aids can be fitted bilaterally from the start in cases of bilateral hearing loss. Therefore, this study excluded those with bilateral hearing loss who preferred bilateral hearing aids.

### Stapedotomy

Stapedotomy was performed at four clinics, two university clinics and two county clinics, that routinely carry out otosclerotic surgery in Sweden. Surgery was performed according to routine at each clinic. All surgeries were performed under general anesthesia. Of the 91 included participants, 83 (91%) underwent outpatient surgery. The removal of the stapes superstructure and fenestration were performed in different ways: only laser (*n* = 19), laser and microdrill (*n* = 69), and only microdrill (*n* = 3). Eighty-five patients received a platinum/teflon piston with a diameter of 0.4 mm, one a platinum/teflon piston with a diameter of 0.6 mm, four a titan piston with a diameter of 0.4 mm, and one a titan piston with a diameter of 0.6 mm.

### Hearing aid fitting

Hearing aids were fitted at four clinics: two university clinics and two county clinics. As the study included clinics from different regions of the country, with each region having its own public procurement process for hearing aids, it was not feasible to ensure that all participants would receive the same type of hearing aid. For the purposes of the study, however, certain general terms were established to reduce variability regarding hearing aid fitting and that were applicable irrespective of the regional directives. All hearing aids were fitted with the same prescription method, the NAL-NL1 [14]. The prescribed gain was verified with a real-ear gain measurement at 50, 65, and 80 dB SPL, although fine-tuning was allowed according to the preferences of the recipient. Only individually-fitted earmolds were used, i.e., no thin tube fittings with silicone domes were included. The hearing aid fitting period consisted of a maximum of three visits.

### Audiometry

Pure tone audiometry was performed before intervention and one year after intervention. Air conduction (AC) thresholds were measured at frequencies 250, 500, 1000, 2000, 3000, 4000, 6000, and 8000 Hz. Bone conduction was measured at 500, 1000, 2000, 3000, and 4000 Hz. Contralateral masking was applied when indicated.

Sound field (SF) audiometry was performed with warble tones presented at 250, 500, 1000, 2000, and 4000 Hz. Speech intelligibility in the SF condition was measured as speech reception threshold (SRT) in noise using the Hagerman matrix sentences [15, 16]. The SRT measurement was performed with a fixed noise presented at 65 dB SPL with an adaptive speech signal, with each measurement consisting of two lists of ten five-word sentences. The result was calculated as the mean signal-to-noise ratio of only the second of the two lists presented. All SF measurements were made in a soundproof booth with a single loudspeaker placed at a distance of 1 m and 0° azimuth. Sound field tests were performed with a 3 M E-A-R Classic Earplug in the contralateral non-treated ear with an attenuation of 30 dB [17].

### Intervention satisfaction and adverse auditory effects

Participants were prompted to fill out a survey at 6 and 12 months. For the purpose of this study the 6 months survey was used only when the 12 month form was missing. The question “Are you satisfied with the surgery/hearing aid?” was used to assess satisfaction with the intervention. The response was given on a five-point Likert scale where a higher number indicates greater satisfaction.

Adverse auditory effects of tinnitus, loudness discomfort, and sound distortion, were assessed with postoperative changes categorized as “better,” “unchanged,” or “worse.” Only symptoms attributed to the intervention ear were analyzed; cases reported bilaterally were considered as involving the intervention ear.

### Statistical analysis

Descriptive statistics were presented as means with standard deviations (SD) for normally distributed data, and as medians with interquartile ranges (IQR) for non-normally distributed data. The Student’s t-test was used for comparisons of continuous variables with normal distributions. For non-normally distributed data, the Wilcoxon signed-rank test was applied. Pairwise comparisons were used for within-group analyses; otherwise, a two-sample method was employed. In cases of multiple comparisons, the Holm correction was applied. For categorical data, Fisher’s exact test was used.

## Results

Table 1 summarizes the participants’ demographics and average hearing parameters before the intervention. There was no significant difference between the two groups regarding sex (*p* = 0.46) or age (*p* = 0.06). Both groups had similar hearing thresholds regarding air and bone conduction for the intervention ear and, conversely, also a similar ABG. Furthermore, the non-intervention contralateral ear also had comparable air conduction thresholds in both groups.

**Table 1:** Demographics and pure tone audiometric average before intervention

	Total	Stapedotomy	Hearing aid	*p*-value*
Participants - N	133	91	42	
Sex (female) - N (%)	79 (59%)	52 (57%)	27 (64%)	0.46
Age year - Median (Range)	46 (22–65)	43 (22–65)	51 (22–65)	0.06
*Intervention ear*				
AC PTA^4^ - Median (IQR)	46.2 (40.0–55.0)	47.5 (41.2–56.2)	44.4 (38.8–52.5)	0.30
BC PTA^4^ - Median (IQR)	18.8 (13.8–25.0)	18.8 (13.8–25.0)	17.5 (14.1–25.0)	0.26
ABG PTA^4^ - Median (IQR)	26.2 (21.2–33.8)	27.5 (21.2–33.8)	25.0 (22.5–31.9)	0.85
*Contralateral ear*				
AC PTA^4^ - Median (IQR)	11.2 (6.2–21.2)	11.2 (6.2–19.4)	11.9 (5.3–21.2)	0.96

*AC* air conduction, *ABG* air bone gap, *BC* bone conduction, *PTA*^4^ pure tone average for 500, 1000, 2000 and 4000 Hz, *IQR* interquartile range

*Wilcoxon signed rank test, Fisher's exact test

### Sound field measurements

**Fig. 2:**
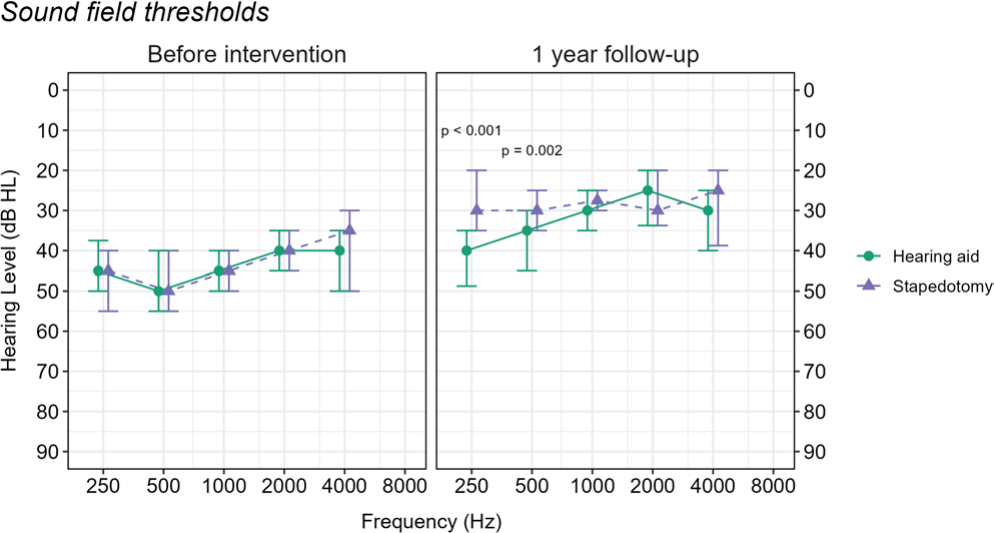
Median and interquartile range for sound field tone audiometry. Comparisons by frequency for the two intervention groups and time points were made with the two-sample Wilcoxon test, applying the Holm correction. Only values *p* < 0.05 are shown in the figure

SF warble tone audiometry showed identical thresholds for the two groups before intervention, Fig. 2. At the one-year follow-up, the stapedotomy group showed significantly greater improvements at 250 and 500 Hz compared to the hearing aid group. For the other tested frequencies, there were no significant differences at follow-up. The average within-group difference across frequencies 500, 1000, 2000, and 4000 Hz before and after intervention was 11.6 dB (SD = 9.2 dB) for the hearing aid group and 15.3 dB (SD = 10.3 dB) for the stapedotomy group, although the between-group difference was not significant according to the two-sample t-test (*p* = 0.08).

**Fig. 3:**
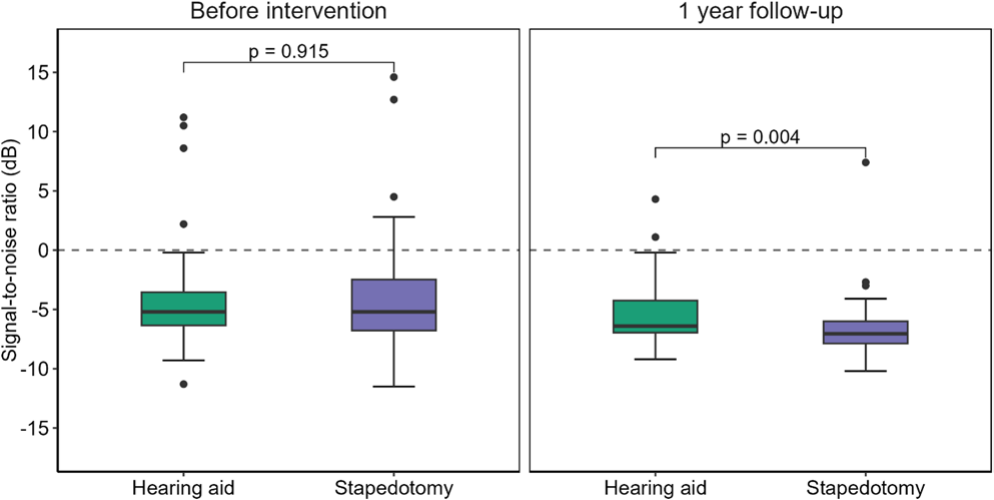
Boxplots for speech reception thresholds measured with the Hagerman matrix sentences in a sound field setting with a fixed speech level of 65 dB SPL and an adaptive noise level. Group comparisons were performed with the two-sample Wilcoxon test

SRT in noise was measured with the Hagerman matrix sentences in an SF setting. Before intervention, the two groups had similar results (*p* = 0.92) Fig. 3. At the one-year follow-up, the stapedotomy group had significantly better results after the intervention compared to the hearing aid group (*p* = 0.004). The within group comparison regarding change before and after the intervention also showed a significant improvement for the stapedotomy group (*p* < 0.001), but not for the hearing aid group (*p* = 0.13).

### Intervention satisfaction and adverse auditory effects

The subjective outcome of satisfaction with the intervention was rated with the question “Are you satisfied with the surgery/hearing aid?”. A total of 87 participants (96%) in the stapedotomy group and 41 (95%) in the hearing aid group responded. Both groups had a high median score, indicating high satisfaction with the intervention received. However, the stapedotomy group had a significantly higher score, with a median of 4 (IQR = 4–5) on the five-point Likert scale, compared to the hearing aid group, with a median of 4 (IQR = 4–4), *p* = 0.019. Notably, the non-variance in the hearing aid group is due to few participants reporting a score other than 4.

As shown in Table 2, tinnitus outcomes did not differ between the stapedotomy and hearing aid groups, with comparable proportions of participants reporting improvement, stability, or worsening (*p* = 0.96). Loudness discomfort was more likely to worsen after stapedotomy than after hearing aid use (*p* = 0.02). No significant differences were found for sound distortion between the groups (*p* = 0.55).

Two surgeries (2%) led to a worsening of the BC PTA^4^ of > 10 dB, with a loss in BC thresholds by 11.3 and 37.5 dB, respectively. The cases were performed by different surgeons at different clinics. No surgery resulted in severe sensorineural hearing loss or deafness.

**Table 2:** Reported changes in tinnitus, loudness discomfort, and sound distortion after Stapedotomy or hearing aid intervention

		Stapedotomy	Hearing aid	*p*-value*
Tinnitus	Better	25 (29%)	12 (29%)	0.96
	Unchanged	24 (28%)	10 (24%)	
	Worse	14 (16%)	8 (20%)	
	Never present	24 (28%)	11 (27%)	
Loudness discomfort	Better	11 (13%)	4 (10%)	**0.02**
	Unchanged	12 (14%)	4 (10%)	
	Worse	34 (39%)	7 (17%)	
	Never present	30 (34%)	26 (63%)	
Sound Distortion	Better	16 (18%)	7 (17%)	0.55
	Unchanged	4 (5%)	3 (7%)	
	Worse	12 (14%)	9 (22%)	
	Never present	55 (63%)	22 (54%)	

*Distributions of outcomes (better, unchanged, worse, never present) were compared between groups using Fisher’s exact test; significant p-values (< 0.05) are in bold.

## Discussion

This prospective, non-randomized intervention study compared hearing outcomes and patient satisfaction between stapedotomy and hearing aid intervention in individuals with previously untreated otosclerosis. The findings demonstrate that both interventions provided measurable benefits, but stapedotomy was associated with significantly better outcomes in speech-in-noise recognition, low-frequency hearing, and patient satisfaction.

One year post-intervention, the stapedotomy group showed a significantly greater improvement in speech reception threshold in noise, compared to the hearing aid group (*p* = 0.004), despite similar baseline performance. Molinier et al. [12] reported comparable results in a shorter two‑month follow-up, where stapedotomy yielded better speech recognition than hearing aids treatment, although in a binaural measurement setting. These findings support the notion that restoring mechanical transmission of sound through surgery may offer advantages in complex listening environments. Even though modern hearing aids have noise reduction algorithms, studies have shown that this feature does not affect speech intelligibility [18].

Stapedotomy resulted in a greater improvement in SF warble tone thresholds at low frequencies (250 and 500 Hz), indicating better low-frequency hearing restoration compared to hearing aid use. This is probably due to the venting of the earmold, which is generally added to reduce the sound of one’s own voice (i.e., the occlusion effect). Even though the vent reduces amplification in low frequencies, this reduction is generally not as large as for closed silicone domes [19]. However, caution is warranted when interpreting SF thresholds measured with hearing aids, as amplification changes with the incoming signal due to non-linear wide dynamic range compression, and the threshold does not inform about audibility at speech level [20].

When elicited, both groups expressed high levels of satisfaction with the intervention, although stapedotomy recipients rated their intervention significantly more favorably (*p* = 0.019). This may reflect the perceived permanence and natural sound quality achieved with surgical correction.

Loudness discomfort occurred significantly more often in the stapedotomy group compared to the hearing aid group (*p* = 0.02), and was the only adverse auditory effect to differ between interventions. In this study 39% of patients undergoing stapedotomy reported increased loudness discomfort, a higher proportion than reported in previous studies. For example, Tan et al. [9] found that 26% reported loud sounds too loud two months after surgery, and Santos et al. [8] noted that the symptom resolved during the first months after surgery. The elevated incidence observed here may relate to differences in follow-up duration or patient characteristics. Additionally, removal of the stapedial tendon eliminates the acoustic reflex, reducing the ear’s natural protection against loud sounds. It has also been suggested that the smaller contact area of the prosthesis at the oval window leads to altered sound force transmission compared to that delivered by the intact stapes, which may affect the perception of loud sounds [8].

In two cases, stapedotomy was associated with a significant worsening of bone conduction thresholds. This observation is consistent with previous studies reporting an increase in sensorineural hearing loss following stapedotomy [21, 22]. Although rare, these findings highlights the potential risk of the procedure.

Importantly, the decision between surgery and hearing aid rehabilitation is multifactorial and must consider patient preferences, medical contraindications, and psychosocial aspects. While stapedotomy offers better auditory outcomes for some, it is not without risk, including potential complications such as vertigo, worsened tinnitus, or, in rare cases, profound hearing loss [5, 7]. Conversely, hearing aids are safer and immediately reversible, although their effectiveness may be limited in restoring natural sound perception, particularly in challenging listening environments. It is also important to note that in cases of prominent mixed hearing loss, stapedotomy may be performed to enhance the effectiveness of a hearing aid, highlighting that these treatments are not necessarily at odds with each other, but can be complementary [3]. Furthermore, in cases of bilateral otosclerosis, the ears are usually operated on in sequence with an interval of at least one year, during which a hearing aid may be used in the unoperated ear [23, 24].

The non-randomized design of this trial is a limitation, as treatment allocation was based on patient preference rather than random allocation. A randomized approach was deemed impractical, as it would have excluded individuals unwilling to undergo surgery, introducing potential selection bias in favor of stapedotomy. Therefore, in this study, we allowed the participants to decide the treatment based on their own preferences, ensuring that all entered the study on equal terms in this respect. This was regarded as the most ethical approach since both treatments are well-established and widely used. Despite this limitation, baseline audiometric characteristics were similar between groups, indicating comparability in terms of audiometric outcomes. While regional differences in hearing aid types and surgical techniques as well as the involvement of multiple surgeons, may have introduced variability, the use of standardized protocols for gain prescription and audiometric assessment helped minimize heterogeneity. Nonetheless, this variability reflects conditions encountered in routine clinical care. This study included a 1-year follow-up period; however, future research is needed investigating the long-term outcomes, particularly of non-surgical treatments for otosclerosis, as evidence in this area remains scarce.

## Conclusion

Stapedotomy may offer better outcomes in low-frequency hearing improvement, speech-in-noise recognition, and patient satisfaction compared to hearing aids in patients with otosclerosis and no prior treatment. However, both interventions are valid and effective, and the choice should be individualized, considering personal preferences and potential risks.

## References

[1] Chole RA, McKenna M (2001) Pathophysiology of otosclerosis. Otol Neurotol 22:249. 10.1097/00129492-200103000-00023 11300278 10.1097/00129492-200103000-00023

[2] Crompton M, Cadge BA, Ziff JL, Mowat AJ, Nash R, Lavy JA, Powell HRF, Aldren CP, Saeed SR, Dawson SJ (2019) The epidemiology of otosclerosis in a British Cohort. Otology & neurotology: official publication of the American otological society. Am Neurotology Soc [and] Eur Acad Otology Neurotology 40:22–30. 10.1097/MAO.0000000000002047 10.1097/MAO.0000000000002047 PMC6314447 30540696

[3] Lancer H, Manickavasagam J, Zaman A, Lancer J (2016) Stapes surgery: a national survey of British otologists. Eur Arch Otorhinolaryngol 273:371–379. 10.1007/s00405-015-3560-6 25711736 10.1007/s00405-015-3560-6

[4] Silva VAR, Pauna HF, Lavinsky J, Guimarães GC, Abrahão NM, Massuda ET, Vianna MF, Ikino CMY, Santos VM, Polanski JF, da Silva MNL, Sampaio ALL, Zanini RVR, Lourençone LFM, Denaro MM, de Calil C, Chone DB, Castilho CT AM (2023) Brazilian society of otology task force – Otosclerosis: evaluation and treatment. Braz J Otorhinolaryngol 89:101303. 10.1016/j.bjorl.2023.101303 37647735 10.1016/j.bjorl.2023.101303 PMC10474207

[5] Strömbäck K, Lundman L, Bjorsne A, Grendin J, Stjernquist-Desatnik A, Dahlin-Redfors Y (2017) Stapes surgery in Sweden: evaluation of a national-based register. European Archives of Oto-Rhino-Laryngology 274:2421–2427. 10.1007/s00405-017-4510-2 28285424 10.1007/s00405-017-4510-2 PMC5420002

[6] Bittermann AJN, Rovers MM, Tange RA, Vincent R, Dreschler WA, Grolman W (2011) Primary stapes surgery in patients with otosclerosis: prediction of postoperative outcome. Archives Otolaryngology–Head Neck Surg 137:780–784. 10.1001/archoto.2011.100 10.1001/archoto.2011.100 21768405

[7] Vincent R, Sperling NM, Oates J, Jindal M (2006) Surgical findings and long-term hearing results in 3,050 stapedotomies for primary otosclerosis: a prospective study with the Otology-Neurotology database. Otol Neurotol 27:S25. 10.1097/01.mao.0000235311.80066.df 16985478 10.1097/01.mao.0000235311.80066.df

[8] Santos M, Rego ÂR, Lino J, Coutinho M, Sousa CA (2021) Hyperacusis and stapes surgery: an observation in fifty patients after stapedotomy. J Otol 16:18–21. 10.1016/j.joto.2020.07.001 33505445 10.1016/j.joto.2020.07.001 PMC7814076

[9] Tan FML, Grolman W, Tange RA, Fokkens WJ (2007) Quality of perceived sound after stapedotomy. Otolaryngol Head Neck Surg 137:443–449. 10.1016/j.otohns.2007.03.038 17765773 10.1016/j.otohns.2007.03.038

[10] Sood S, Strachan DR, Tsikoudas A, Stables GI (2002) Allergic otitis externa. Clin Otolaryngol Allied Sci 27:233–236. 10.1046/j.1365-2273.2002.00584.x 12169122 10.1046/j.1365-2273.2002.00584.x

[11] Ruusuvuori JE, Aaltonen T, Koskela I, Ranta J, Lonka E, Salmenlinna I, Laakso M (2021) Studies on stigma regarding hearing impairment and hearing aid use among adults of working age: a scoping review. Disabil Rehabil 43:436–446. 10.1080/09638288.2019.1622798 31177867 10.1080/09638288.2019.1622798

[12] Molinier C-E, Gallois Y, Deguine O, Iversenc G, Vales O, Taoui S, Lepage B, Fraysse B, Marx M (2022) Stapedotomy versus hearing aids in the management of conductive hearing loss caused by otosclerosis: A prospective comparative study. Otology Neurotology 43:773–780. 10.1097/MAO.0000000000003585 35878633 10.1097/MAO.0000000000003585

[13] Committee on Hearing and Equilibrium (1995) Committee on hearing and equilibrium guidelines for the evaluation of results of treatment of conductive hearing loss. Otolaryngology–Head Neck Surg 113:186–187. 10.1016/S0194-5998(95)70103-6 10.1016/S0194-5998(95)70103-6 7675477

[14] Byrne D, Dillon H, Ching T, Katsch R, Keidser G (2001) NAL-NL1 procedure for fitting nonlinear hearing aids: characteristics and comparisons with other procedures. J Am Acad Audiol 12:37–51 11214977

[15] Hagerman B (1982) Sentences for testing speech intelligibility in noise. Scand Audiol 11:79–87. 10.3109/01050398209076203 7178810 10.3109/01050398209076203

[16] Hagerman B, Kinnefors C (1995) Efficient adaptive methods for measuring speech reception threshold in quiet and in noise. Scand Audiol 24:71–77. 10.3109/01050399509042213 7761803 10.3109/01050399509042213

[17] 3 M (2023) 3 M E-A-R Classic Earplugs - Technical datasheet. [Online] https://multimedia.3m.com/mws/media/2075314O/3m-e-a-r-classic-earplugs-technical-datasheet-nl-notified-body.pdf. Accessed 4 Jun 2025

[18] Lakshmi MSK, Rout A, O’Donoghue CR (2021) A systematic review and meta-analysis of digital noise reduction hearing aids in adults. Disabil Rehabil Assist Technol 16:120–129. 10.1080/17483107.2019.1642394 31502900 10.1080/17483107.2019.1642394

[19] Dillon H (2012) Hearing aid earmolds, earshells and coupling systems. Hearing aids, 2nd edn. Boomerang, New York : Thieme, pp 127–169

[20] Kuk F, Ludvigsen C (2003) Reconsidering the concept of the aided threshold for nonlinear hearing AIDS. Trends in Amplification 7:77–97. 10.1177/108471380300700302 15004648 10.1177/108471380300700302 PMC4168919

[21] House HP, Hansen MR, Al Dakhail AAA, House JW (2002) Stapedectomy versus stapedotomy: comparison of results with long-term follow-up. Laryngoscope 112:2046–2050. 10.1097/00005537-200211000-00025 12439178 10.1097/00005537-200211000-00025

[22] Fang Y, Zhang K, Ersbo JH, Chen B (2021) The impact of the frequency-specific preoperative sensorineural hearing loss to postoperative overclosure of bone conduction in stapedotomy. Otol Neurotol 42:1314–1322. 10.1097/MAO.0000000000003320 34528921 10.1097/MAO.0000000000003320

[23] Luca M, Massimilla EA, Americo M, Michele N, Donadio A, Gaetano M (2023) Stapes Surgery in Far-Advanced Otosclerosis. Ear Nose Throat J 102:611–615. 10.1177/01455613211013093 33971751 10.1177/01455613211013093

[24] Redfors YD, Hellgren J, Möller C (2013) Hearing-aid use and benefit: a long-term follow-up in patients undergoing surgery for otosclerosis. Int J Audiol 52:194–199. 10.3109/14992027.2012.754957 23336672 10.3109/14992027.2012.754957

